# Regional cortical perfusion increases induced by a 6-month endurance training in young sedentary adults

**DOI:** 10.3389/fnagi.2022.951022

**Published:** 2022-08-09

**Authors:** Neeraj Upadhyay, Theresa Schörkmaier, Angelika Maurer, Jannik Claus, Lukas Scheef, Marcel Daamen, Jason A. Martin, Rüdiger Stirnberg, Alexander Radbruch, Ulrike Attenberger, Tony Stöcker, Henning Boecker

**Affiliations:** ^1^Clinical Functional Imaging Group, Department of Diagnostic and Interventional Radiology, University Hospital Bonn, Bonn, Germany; ^2^German Center for Neurodegenerative Diseases, Bonn, Germany; ^3^Department of Neuroradiology, University Hospital Bonn, Bonn, Germany; ^4^Department of Diagnostic and Interventional Radiology, University Hospital Bonn, Bonn, Germany; ^5^Department of Physics and Astronomy, University of Bonn, Bonn, Germany

**Keywords:** maximum oxygen uptake, endurance training, perfusion, pseudo-continuous arterial spin labeling, brain perfusion, CBF

## Abstract

Physical inactivity is documented as a health risk factor for chronic diseases, accelerated aging, and cognitive impairment. Physical exercise, on the other hand, plays an important role in healthy aging by promoting positive muscular, cardiovascular, and central nervous system adaptions. Prior studies on the effects of exercise training on cerebral perfusion have focused largely on elderly cohorts or patient cohorts, while perfusion effects of exercise training in young sedentary adults have not yet been fully assessed. Therefore, the present study examined the physiological consequence of a 6-month endurance exercise training on brain perfusion in 28 young sedentary adults randomly assigned to an intervention group (IG; regular physical exercise) or a control group (CG; without physical exercise). The IG performed an extensive running interval training three times per week over 6 months. Performance diagnostics and MRI were performed every 2 months, and training intensity was adapted individually. Brain perfusion measurements with pseudo-continuous arterial spin labeling were analyzed using the standard Oxford ASL pipeline. A significant interaction effect between group and time was found for right superior temporal gyrus (STG) perfusion, driven by an increase in the IG and a decrease in the CG. Furthermore, a significant time effect was observed in the right middle occipital region in the IG only. Perfusion increases in the right STG, in the ventral striatum, and in primary motor areas were significantly associated with increases in maximum oxygen uptake (VO_2max_). Overall, this study identified region-specific increases in local perfusion in a cohort of young adults that partly correlated with individual performance increases, hence, suggesting exercise dose dependency. Respective adaptations in brain perfusion are discussed in the context of physical exercise-induced vascular plasticity.

## Introduction

There is a broad consensus across disciplines that a sedentary lifestyle is an important risk factor for chronic diseases (Booth et al., 2012), accelerated aging (Mattson and Arumugam, 2018), and cognitive impairment (Steinberg et al., 2015). Physical exercise (PE) plays an important role in current concepts of “healthy aging” (Bull et al., 2020). Effects on the central nervous system are promoted by exercise-induced neurogenesis, synaptic plasticity, angiogenesis, and improved cerebral blood flow (CBF)/metabolism (Tarumi and Zhang, 2015; Vivar and Van Praag, 2017). Notably, these mechanisms impact the brain areas typically affected by aging, thereby maintaining or even improving cognitive function (Hillman et al., 2008; Erickson et al., 2011).

The human brain consumes ∼20% of total oxygen consumption at rest, and the brain function critically depends on continuous delivery of oxygen and metabolic nutrients (Smith and Ainslie, 2017). Regional brain perfusion is tightly coupled to regional cerebral metabolism (Chen et al., 2015). Brain perfusion and metabolism are inversely associated with age, and CBF is typically compromised in neurological diseases (Chen et al., 2015). On the other hand, intact cortical perfusion is considered a surrogate for an uncompromised tissue delivery of metabolites through cerebral microvessels that influence neuronal energy metabolism (Watts et al., 2018). PE is known to trigger angiogenesis *via* a complex interplay of growth factors, particularly the vascular endothelial growth factor (VEGF), and various receptors and signaling pathways that serve as stimuli for capillary growth (Egginton, 2009). Subsequent adaptations of CBF are necessary for meeting the changing metabolic demands associated with repeated exercise challenges (Ogoh and Ainslie, 2009), as well as non-acute or long-term neuroplastic changes induced by PE (Hillman et al., 2008; Erickson et al., 2011).

While structural and functional brain plasticity have been thoroughly investigated in numerous previous exercise imaging studies (for meta-analyses see Ji et al., 2021; Yu et al., 2021), the effects of PE on resting levels of global and regional brain perfusion have not yet been fully elucidated (Lehmann et al., 2020). Moreover, the existing PE interventions so far were exclusively conducted in older healthy cohorts (Burdette et al., 2010; Chapman et al., 2013; Flodin et al., 2017; Kleinloog et al., 2019) or patient populations (Robertson et al., 2017; Alfini et al., 2019; Thomas et al., 2020), but not in healthy young adults.

Given that recommendations on brain health and disease prevention are shifting to earlier ages (Ortega et al., 2008; Wen et al., 2011; Lin et al., 2015), our study aimed at determining whether exercise training for a 6-month duration significantly increases CBF in younger cohorts also. Optimal brain perfusion is considered to be an important component of “brain health” responsible for an adequate supply of nutrients and oxygen. In particular, we were interested where and when CBF changes would occur in the brain. For this purpose, we designed a randomized controlled intervention trial testing the effects of PE training over a 6-month-period on CBF in sedentary adults aged 20–35 years. We used whole-brain pseudo-continuous arterial spin labeling (pCASL) to measure PE-induced CBF increases and to relate changes in CBF to individual performance increases, as assessed in repeated graded exercise tests. Based on previous literature, we hypothesized that PE-induced perfusion increases in the following areas: (i) in the temporal lobe where PE interventions of 3 (Maass et al., 2015) or 4 (Burdette et al., 2010) months duration have highlighted hippocampal perfusion increases in healthy elderly cohorts (Pereira et al., 2007; Burdette et al., 2010; Maass et al., 2015); (ii) in the anterior cingulate cortex where other randomized PE studies of 12-weeks duration reported CBF increases in the elderly subjects (Chapman et al., 2013; Kleinloog et al., 2019); (iii) in areas of the frontal cortex which show exercise-induced plasticity (Zheng et al., 2019; Northey et al., 2020; Tari et al., 2020; Zhu et al., 2021); and finally (iv) in the primary motor cortex where structural plasticity was reported in endurance runners (Cao et al., 2021).

## Materials and methods

### Subjects

Men and women in the age group of 18–35 years were recruited *via* flyers distributed at the local university and on social media. Before the study inclusion, we verified that subjects had a sedentary lifestyle and a poor to fair fitness level. Subjects with prior history as competitive athletes (according to a custom questionnaire on different life spans, namely, up to 12 years of age, 13–18 years of age, after 18 years of age until inclusion) or regular physical exercise training in the last 2 years preceding this study (according to a custom questionnaire on frequency, time, and type of exercise) were excluded. As an add-on, the International Physical Activity Questionnaire (IPAQ) assessed participants’ general physical activity behavior, including leisure and work-associated activities, during the previous 7 days.

Eligible subjects did not meet the evidence-based recommendations for frequency, intensity, time, and type of exercise for developing and maintaining cardiorespiratory, musculoskeletal, and neuromotor fitness regarding ACSM Position Stand (Garber et al., 2011). Physical activities like slow walking or other incidental activities did not lead to exclusion.

To determine that the participants were healthy and to exclude major physical health risks for subsequent exercise tests and trainings, a medical healthcare check was done before randomization into the study in all subjects. This included an anamnestic questionnaire, auscultation of lung and heart, and a 12-channel resting electrocardiogram, as well as questionnaires regarding neurologic and psychiatric diseases (current or in the past). Based on these tests, the sedentary participants were classified as being eligible for inclusion into a randomized exercise intervention. Screening questionnaires (Mini International Neuropsychiatric Interview (M.I.N.I, german version 5.0.0; Sheehan et al., 1998), trait anxiety of the State-Trait-Anxiety Inventory (STAI; Spielberger et al., 1983), and Beck Depression Inventory (BDI-I; Hautzinger et al., 1994) provided no indications for neurological or psychological diseases. MRI-related exclusion criteria were pregnancy, claustrophobia, non-removable metal, and tattoos exceeding a certain size (>20 cm or covering > 5% body surface or in head or neck area). All subjects were informed about the procedure and protocols and signed a declaration of agreement before inclusion in the study. The procedures were approved by the local ethics committee (Ethikkommission an der Medizinischen Fakultät der Rheinischen Friedrich-Wilhelms-Universität Bonn: Nr. 370/15) and were conducted according to the Declaration of Helsinki. The study was registered in the DRKS – German Clinical Trials Register (clinical trial registration number DRKS00021460).

### Experimental design

**FIGURE 1:**
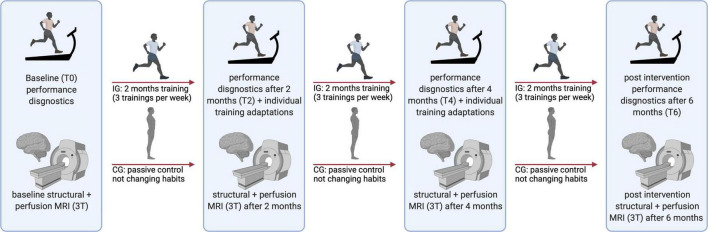
Flow diagram of the study showing examinations at each time point (T0, T2, T4, and T6). 3T, 3 Tesla; MRI, magnetic resonance imaging; CG, control group; IG, intervention group. Figure was created with BioRender.com.

This study is a sub-analysis within the “RUNSTUD” study (Maurer et al., 2020). To test the influence of an extensive interval training over 6 months on brain structure and function, all subjects completed an extensive battery of neuropsychological, 3T and 7T MRI, and pain assessments before (timepoint at baseline: T0) and every 2 months (2, 4, and 6 months; timepoints T2, T4, and T6) after being randomized into an intervention group (IG) or control group (CG) (Figure 1).

To exclude major physical health risks for subsequent exercise tests and trainings, a medical healthcare check was done before inclusion in the study in all subjects. This included an anamnestic questionnaire, auscultation of the lungs and heart, and a 12-channel resting electrocardiogram. To test the influence of an extensive interval training over 6 months on brain perfusion, all subjects (CG and IG) performed a maximal graded exercise test on the treadmill to determine physical fitness. Subjects were not allowed to perform strenuous exercise or drink alcohol 24 h before the examination day and were instructed to refrain from caffeine (and for exercise testing: food) at least 2 h before the testing sessions.

### Incremental exercise test

The incremental exercise test was performed on a treadmill (PPS S70, Woodway GmBH, Weil am Rhein, Germany) with an initial running speed of 6 km h^–1^ and an incline set to 1%. Running speed increased in 1 km h^–1^ increments every 3 min until voluntary exhaustion to determine maximal oxygen uptake (VO_2max_) and maximal heart rate (HR_max_). Oxygen uptake (VO_2_), metabolic respiratory quotient (respiratory exchange ratio, RER), and heart rate (HR) were continuously measured during the test (Cortex meta-analyzer 3B, Leipzig, Germany, and Polar Electro A360, Kempele, Finland). Capillary blood lactate samples were taken from the fingertip in the last 15 s of each step. To determine lactate concentration, 20 μl of capillary blood was directly mixed with 1 mL of the EBIO plus system hemolysis solution and analyzed amperometric-enzymatically using EBIOplus (EKF Diagnostic Sales, Magdeburg, Germany). At the same time points, the rating of perceived exertion (RPE) was assessed using the 6–20 points Borg scale (Borg, 1970). Exhaustion was considered with the attainment of at least two of the following criteria: leveling off in VO_2_, RER ≥ 1.10, high levels of blood lactate (BLa) (≥8 mmol L^–1^), a perceived rate of exertion of ≥18, and/or a HR of ±10 bpm of age-predicted maximum (220 – age) (Midgley et al., 2007). The relative VO_2max_ was defined as the highest 30 s moving average of VO_2_ divided by body mass (mL min^–1^ kg^–1^).

### Endurance exercise intervention

Throughout the 6-month running intervention phase, subjects of the intervention group completed three running sessions per week lasting 25–45 min, consisting of two supervised exercise sessions on the treadmill, one in the laboratory and one at home on flat terrain. This resulted in a total of 78 training sessions of which 60.0 ± 11.1 (77 ± 14%) were performed on average. An activity tracker was used to track subjects’ training data of the home trainings in the IG and to rule out deliberate physical activity in the CG.

The endurance exercise training of the intervention group was designed as an extensive interval training with 3–5-min intervals at 75–80% of HR_max_ and 3–5 min of active recovery, with six to eight repetitions. To ensure a steady progression of physical adaptations, exercise intensity was adapted individually according to the results of each performance test (2 and 4 months after baseline) (Figure 1).

Subjects in the CG were instructed to stick to their usual lifestyle, refrain from any kind of exercise, and continue their normal dietary and physical activity practices throughout the study.

### Magnetic resonance imaging acquisition

The magnetic resonance imaging (MRI) data were acquired using a 3T Siemens Magnetom Skyra scanner. Perfusion data was acquired using an in-house developed 3D pCASL sequence (Boland et al., 2018) with TR = 4,130 ms; TE = 21.50 ms; FOV = 224 mm; slice thickness = 3.5 mm; bolus length = 1,800 ms, post-labeling duration = 1,800 ms, and total duration = 5 min:48 s.

We also acquired structural data sets using a custom T1-weighted MPRAGE sequence (Brenner et al., 2014), which employs threefold parallel imaging acceleration with CAIPIRINHA (Breuer et al., 2006) and elliptical sampling (Bernstein et al., 2001). The sequence parameters were as follows: TR = 2,500 ms, TE = 5 ms, TI = 1,100 ms, flip angle = 7°; FOV = 256 mm; voxel size = 1 mm^3^ × 1 mm^3^ × 1 mm^3^, 192 slices, scan duration 2 min:53 s.

### Data analysis

#### Physiological data

A linear mixed effect (LME) model was performed to investigate the fitness measure VO_2max_ among the groups (IG and CG) at the different time points (T0, T2, T4, and T6), as well as their interaction (group × time). LME models allow to overcome the issue of missing data values across time points (Ibrahim and Molenberghs, 2009). In this model, we included age and sex as fixed effects and a random intercept to account for random individual-level effects. *Post hoc* comparisons were performed using an LME model with time as the categorical variable using the “emmeans” package (Searle et al., 1980). The above models were obtained from the package lme4 (Bates et al., 2015) in the open access platform R, version 4.1.0 (R Core Team, 2013).

#### Perfusion data

Pseudo-continuous arterial spin labeling data were analyzed using the standard Oxford ASL pipeline available in the FSL toolbox^1^ (Chappell et al., 2009). Voxel-wise CBF maps were created after correcting for motion and registering to the native gray matter and white matter segmentation maps. Additionally, partial volume corrections were performed to avoid the effect of overlapping CBF values between different tissues. Images were quantified using the default parameters recommended in ASL white paper (Alsop et al., 2015) with the following parameters: longitudinal relaxation time of blood (T1b) = 1,650 ms, longitudinal relaxation time of tissue (T1tissue) = 1,300 ms, gray matter (GM) threshold = 0.8. The resulting GM CBF maps were further normalized to the global mean GM perfusion to create relative GM CBF maps correcting for any time-point-related bias (Yang et al., 2020).

The statistical analysis of these relative GM CBF maps was performed on a voxel by voxel level, including all time points, using the linear mixed-effects model (3dLMEr) (Chen et al., 2013) implemented in the AFNI toolbox (AFNI version: AFNI_21.3.02; 3dLMEr version 0.1.4). Age and sex were included as fixed effects and a random intercept to account for random individual-level effects. Significance was considered at a cluster-defining threshold of *p* < 0.001 (uncorrected) and an FWE-corrected threshold of *p* < 0.05. Later, we performed *post hoc* tests using emmeans package (Searle et al., 2012) implemented in R (version 4.1.0, R Core Team, 2013) and the multivariate *t*-distribution method to adjust *p*-values. The degrees-of-freedom method was Kenward–Roger (Kenward and Roger, 1997). Results of *post hoc* tests are reported with Cohen’s *d* as the effect size (Cohen, 1992).

Furthermore, for regression analysis, the relationship between relative CBF and VO_2max_ was assessed using the above-mentioned 3dLMEr model, with age and sex as covariates, and also using a cluster-defining threshold of *p* < 0.001 (uncorrected) and an FWE-corrected threshold of *p* < 0.05. The Pearson correlation coefficient was calculated by extracting the beta values from the corresponding clusters and implementing the correlation with VO_2max_ in the open access platform R, version 4.1.0 (R Core Team, 2013). The Talairach atlas available in AFNI (Lancaster et al., 2000) was used for labeling the significant results. For reporting and visualization, the statistical maps were transformed into MNI space and overlaid onto the MNI template using MRIcroGL.^2^ Finally, we conducted a targeted analysis for a hippocampal region of interest (ROI) created using the Harvard-Oxford-subcortical structural atlas (Frazier et al., 2005). Following this, we applied the abovementioned LME model to assess changes over time within and between groups.

## Results

**FIGURE 2:**
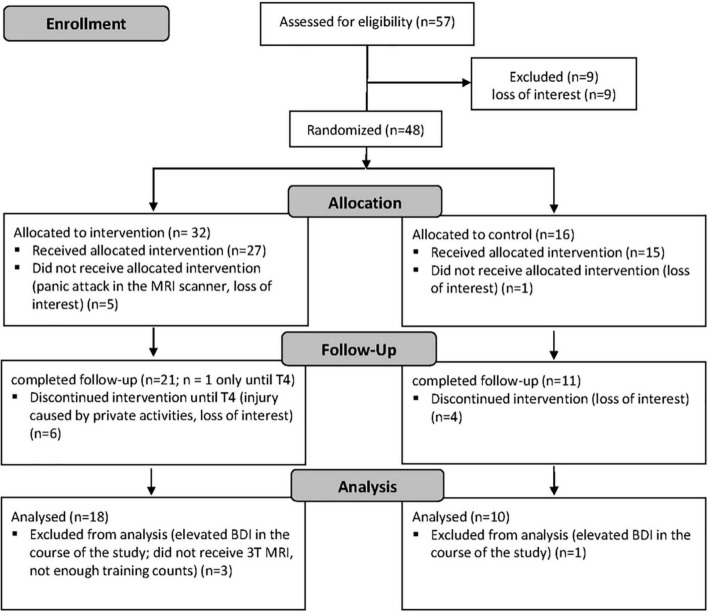
Flow chart diagram. T4, examination timepoint after 4 months.

In total, 57 subjects with a sedentary lifestyle were assessed for eligibility after giving informed consent (Figure 2). Of these, nine were excluded due to loss of interest. Of the remaining 48 subjects, 32 were randomized to the intervention group (IG) and 16 to the control group (CG), respectively (randomization 2:1 IG and CG). Five of the subjects allocated to the IG did not receive the intervention due to panic attacks in the MRI or loss of interest, and another six subjects discontinued the intervention due to injury and loss of interest, leaving a total of 21 subjects in the IG who completed the follow-up. Of the 16 subjects allocated to the CG, one subject did not receive the intervention due to loss of interest, and four dropped out during the intervention due to loss of interest, leaving a total of 11 control subjects who completed the follow-up. After the final exclusion of three further subjects from the IG and one subject from the CG for reasons that became apparent during the intervention, the final dataset consisted of 28 subjects, including 18 in the IG and 10 in the CG. Subject characteristics are given in Table 1. According to the ACSM fitness categories VO_2max_ values below 35.2 mL min^–1^ kg^–1^ in women and below 44.6 mL min^–1^ kg^–1^ in men are defined as poor and, respectively, fair physical fitness. Mean VO_2max_ at T0 in all male subjects was 43.5 ± 3.7 mL min^–1^ kg^–1^ and 32.7 mL min^–1^ kg^–1^ in the female subjects.

**TABLE 1:** Subject characteristics.

	Intervention group (*n* = 18) mean ± SD	Control group (*n* = 10) mean ± SD	*P*-value
Age (years)	23.9 ± 3.9	23.7 ± 4.2	0.80
Gender (M/F)	7/11	6/4	0.50
Height (cm)	173.6 ± 12.1	176.9 ± 7.9	0.45
Weight (kg)	69.9 ± 15.1	71.2 ± 14.1	0.82
EHI	74.2 ± 16.2	79.5 ± 13.3	0.39
WST	107.0 ± 9.9	107.3 ± 8.8	0.94
**VO_2max_ (ml⋅min^–1^ kg^–1^)**			
T0	38.5 ± 3.4	41.7 ± 7.5	0.663
T2	41.0 ± 4.1	39.1 ± 7.2	0.585
T4	42.9 ± 5.4	41.1 ± 8.4	0.164
T6	42.4 ± 5.0	40.3 ± 7.4	0.086
**HR_rest_ (bpm)**			
T0	80.5 ± 14.2	75.6 ± 10.2	0.741
T2	76.9 ± 8.4	71.4 ± 11.4	0.768
T4	72.7 ± 10.9	80.8 ± 9.2	0.239
T6	75.5 ± 11.9	72.1 ± 11.6	0.964
**BP_Diastolic_ (mmHg)**			
T0	74.3 ± 7.3	76.0 ± 11.5	0.956
T2	76.0 ± 6.4	68.3 ± 6.1	0.325
T4	77.5 ± 7.1	71.6 ± 9.0	0.435
T6	78.4 ± 9.6	74.0 ± 6.2	0.649
**BP_systolic_ (mmHg)**			
T0	118.8 ± 10.5	119.0 ± 13.6	0.999
T2	118.0 ± 10.1	118.6 ± 12.5	0.999
T4	115.8 ± 12.4	116.9 ± 8.4	0.966
T6	117.0 ± 9.38	116.6 ± 11.8	0.999
BDI	2.6 ± 3.4^a^	1.4 ± 1.5	0.300
STAI trait	33.9 ± 9.3	31.1 ± 5.8	0.400

^a^Two missing values. bpm, beats per minute; BDI, Beck Depression Inventory 1 (score ≤9: no depression); cm, centimeter; EHI, Edinburgh Handedness Inventory; F, female; HR_max_, maximum heart rate; kg, kilogram; M, male; min, minute; ml, milliliter; SD, standard deviation; STAI, State Trait Anxiety Inventory (range: 20 = not being afraid up to 80 = maximum intensity of anxiety); VO_2max_, maximum oxygen uptake; WST, Wortschatztest. p-value shows differences between groups.

### Relative maximum oxygen uptake (VO_2max_)

The LME model with time as the random slope and random intercept revealed an increase in VO_2max_ in the IG over time. The VO_2max_ showed a significant time effect [F(1, 22.89) = 9.39, *p* = 0.006] and a significant group × time interaction [F(1, 22.85) = 29.67, *p* < 0.001]. There was no significant group effect [F(1, 22.47) = 0.42, *p* = 0.523]. Additionally, a significant effect of sex [F(1, 22.38) = 14.93, *p* < 0.001], driven by higher VO_2max_ values in men than in women was found. Moreover, the covariate age showed no significant impact on the VO_2max_ [F(1, 41.39) = 0.07, *p* = 0.787]. Results of *post hoc* tests within and between IG and CG at each time point are summarized in Table 2.

**TABLE 2:** Results of ***post hoc*** test of the VO_2max_ within and between IG and CG.

Contrast	*t*	*P*-value	Cohen’s *d*
**IG**			
T0 vs. T2	−4.13	<0.001	−1.46
T0 vs. T4	−7.37	<0.001	−2.61
T0 vs. T6	−6.75	<0.001	−2.50
**CG**			
T0 vs. T2	1.09	0.697	−0.55
T0 vs. T4	0.84	0.834	−0.39
T0 vs. T6	1.94	0.219	−0.87
**IG vs. CG**			
T0	−0.78	0.682	−0.90
T2	0.94	0.570	1.11
T4	1.80	0.158	2.09
T6	2.11	0.089	2.46

Post hoc tests comparing different timepoints within group (intervention or control) and comparisons between group for each timepoint (intervention vs. control). All results were controlled for age and sex. CG, control group (n = 10); IG, intervention group [(n = 18) n = 16 at T6]; t, t-statistic; T0, T2, T4, and T6, examination timepoint after 0, 2, 4, and 6 months; VO_2max_, maximum oxygen uptake.

### Comparison of the relative gray matter perfusion

**FIGURE 3:**
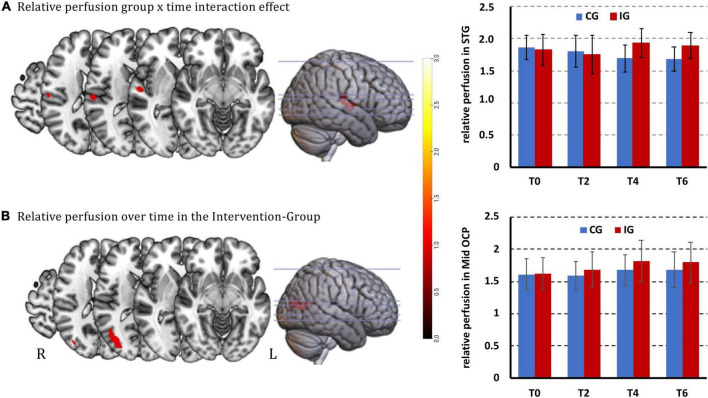
Significant relative perfusion changes [*p* < 0.001 (uncorrected), cluster level corrected at alpha = 0.05 with cluster size > 41 voxel for cluster-wise FWE]. **(A)** Increased group × time interaction effect in the right superior temporal gyrus and bar plots of beta values with standard deviations; **(B)** increased relative perfusion over time in the IG in the right middle occipital region. Clusters are overlaid on the structural MNI template of the brain and bar plots of beta values with standard deviations. The color bar from dark red to light yellow indicates the increasing relative GM CBF values.

Analysis using the 3dLMEr model revealed a significant interaction effect (group × time; cluster size > 41 voxels) for relative GM perfusion in the right superior temporal gyrus (STG) [peak voxel (MNI) at *x*, *y*, *z* = 56.2, −14.9, 7.9]. This interaction was driven by a relative perfusion increase in the IG and a relative perfusion decrease in the CG over time (Figure 3A). Furthermore, a significant time effect was observed in the right middle occipital region (*x*, *y*, *z* = 38.5, −73.9, 13.8) in the IG group, namely an increase in relative perfusion (Figure 3B). The CG did not show significant changes over time in the 3dLMEr model.

*Post hoc* paired *t*-tests of the perfusion changes in right STG over time within the IG revealed a strong trend (after correction for multiple comparisons) for perfusion increase from T0 to T4, but no significance between T0 and T2 and T0 to T6. *Post hoc* paired *t*-tests in CG showed a significant perfusion decrease from T0 to T4 and from T0 to T6, but no significance between T0 and T2. Further, group comparisons showed significantly higher perfusion in the IG group at T4 and T6, but no significant difference at T0 and T2 when compared to the CG.

*Post hoc* paired *t*-test of the perfusion changes in the right middle occipital region showed significant increases from T0 to T4 and from T0 to T6 in the IG.

All detailed statistics are summarized in Table 3.

**TABLE 3:** Results of *post hoc* tests of the relative gray matter (GM) perfusion analysis.

Contrast	*t*	*P*-value	Cohen’s *d*
**Right superior temporal gyrus**			
**IG**			
T0 vs. T2	1.648	0.358	0.549
T0 vs. T4	−*2*.*551*	0.06	−*0*.*85*
T0 vs. T6	1.807	0.278	−*0*.*626*
**CG**			
T0 vs. T2	1.06	0.715	0.474
T0 vs. T4	2.939	0.022	1.314
T0 vs. T6	3.048	0.016	1.363
**IG vs. CG**			
T0	−*0*.*159*	0.999	0.11
T2	−*0*.*268*	0.994	0.185
T4	2.972	0.015	2.054
T6	2.692	0.03	1.879
**Right_middle occipital**			
**IG**			
T0 vs. T2	−*1*.*129*	0.673	−*0*.*376*
T0 vs. T4	−*4*.*343*	<*0*.*001*	−*1*.*448*
T0 vs. T6	−*4*.*134*	<*0*.*001*	−*1*.*434*
**CG**			
T0 vs. T2	0.456	0.968	0.204
T0 vs. T4	–1.095	0.693	–0.490
T0 vs. T6	–1.126	0.674	-0.504
**IG vs. CG**			
T0	–0.336	0.976	–0.295
T2	–0.998	0.580	–0.874
T4	–1.429	0.322	–1.252
T6	–1.389	0.343	–1.224

Post hoc tests comparing different timepoints within group (intervention or control) and comparisons between group for each timepoint (intervention vs. control). All results were controlled for age and sex. CG, control group (n = 10); IG, intervention group [(n = 18) n = 16 at T6]; t, t-statistic; T0, T2, T4, and T6, examination timepoint after 0, 2, 4, and 6 months.

### Regression analysis

**FIGURE 4:**
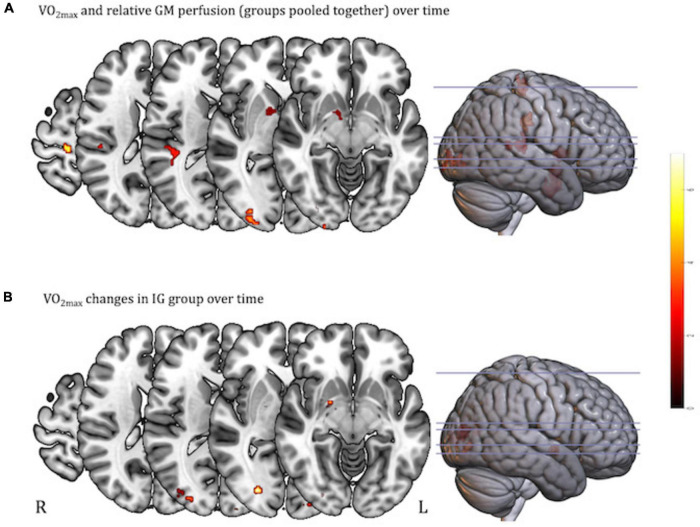
Regression analysis between VO_2max_ and relative gray matter (GM) perfusion using the 3dlmer model in AFNI [*p* < 0.001 (uncorrected), cluster corrected at alpha = 0.05 with cluster size (>42 voxel) for FWE]. **(A)** Higher VO_2max_ associated with higher relative GM CBF is shown in right STG, right middle occipital region, right precentral gyrus, and right ventral striatum (groups pooled together); **(B)** regression analysis for IG only showing higher VO_2max_ associated with higher relative GM CBF in the bilateral middle occipital region, as well as a trend in the right ventral striatum (displayed at the more liberal threshold of *p* < 0.005, uncorrected, >10 voxel). Clusters are overlaid on the structural MNI template of the brain. The color bar from dark red to light yellow indicates the increasing strength of the correlation between VO_2max_ and relative GM perfusion values.

After pooling all subjects and time-points, regression analysis revealed that higher VO_2max_ was associated with higher relative GM CBF in the right STG (*x*, *y*, *z* = 43, −31.9, 13.0; Supplementary Figure 1), the right middle occipital region (*x*, *y*, *z* = 30.4, −95.3, −1.0; Supplementary Figure 1), the right precentral gyrus (*x*, *y*, *z* = 15.7, −28.9, 66.9; Supplementary Figure 1), and the right ventral striatum (*x*, *y*, *z* = 12.7, 5.0, 4.2; Supplementary Figure 1), including the right lentiform nucleus (cluster size > 42 voxels; Figure 4A). To assess the contribution of each group separately, *post hoc* analysis assessed the relationship between VO_2max_ and relative CBF in the IG and CG separately. Therefore, the regression was restricted to those areas showing significance in the above regression analysis (at an inclusive masking threshold of *p* < 0.01, uncorrected) of both groups pooled. Only the IG showed a significant relation between VO_2max_ and relative GM CBF located in the right middle occipital region (*x*, *y*, *z* = 29, −87.9, 12.3; Supplementary Figure 1) and the left middle occipital region (*x*, *y*, *z* = −43.3, −73.9, 14.5; Supplementary Figure 1), as well as a trend in the right ventral striatum (*x*, *y*, *z* = 20.1, −2.4, −4.7; *r* = 0.19; *p* < 0.005 uncorrected, >10 voxel; Figure 4B and Supplementary Figure 1).

No significant changes were observed in relative CBF in the left or right hippocampus ROI. Neither group × time interaction nor the group and time main effects were significant.

## Discussion

This randomized exercise intervention study provides novel evidence that 6 months of extensive interval training (three weekly training sessions of 25–45 min each) over a 6-month period leads to significant increases in regional cortical perfusion in young and previously sedentary adults. Specifically, an interaction effect between group and time was observed in the right STG, which was driven by an increase in perfusion over time in the IG and a significant decrease within the CG. These effects were significantly different between groups after 4 and 6 months of PE training. In addition, the perfusion increase in the right STG was significantly associated with the extent of improvement in physical performance (individual changes in VO_2max_), suggesting dose-dependent training effects on cerebral perfusion. Furthermore, positive associations between fitness changes and local perfusion enhancements were observed in the bilateral occipital lobe, the right precentral gyrus, and the ventral striatum.

To our knowledge, this is the first randomized intervention investigating long-term PE-induced perfusion changes as a marker of vascular plasticity in healthy young adults. As yet, the existing intervention studies reporting perfusion changes related to long-term exercise had been conducted exclusively in older healthy cohorts (Burdette et al., 2010; Chapman et al., 2013; Flodin et al., 2017; Kleinloog et al., 2019) or in patient populations (Robertson et al., 2017; Alfini et al., 2019; Thomas et al., 2020).

The temporal lobe is typically affected by age-related atrophy (Jack et al., 1998) at the levels of the cortex, the hippocampus, the temporal horn, and the white matter (Bigler et al., 2002). Temporal lobe atrophy is thought to contribute to the cognitive impairments in memory that occur in older age (Burke and Barnes, 2006). Results of enhanced perfusion due to PE point to a recent cross-sectional study in which VO_2max_ was positively associated with cortical thickness in the left STG (Olivo et al., 2021). On a meta-analytic level, findings on structural plasticity in the STG associated with physical activity or exercise have been reported using the activation likelihood estimation (ALE) method, where 412 healthy older subjects pooled from nine randomized controlled trials showed significant structural increases in this area (Zheng et al., 2019). Notably, our study showed an association between temporal lobe perfusion and VO_2max_.

We would like to speculate that the interaction effect in the right STG is indeed related to the exercise intervention and the induction of vascular plasticity in the IG only. A cross-sectional MR angiography study found elevated small-caliber vessels in the cortex of aerobically active compared to low-activity healthy subjects (Bullitt et al., 2009). The same study also reported reduced brain-vessel tortuosity in the aerobically active subjects. This is in line with our interpretation that the observed interaction effect could stem from such fundamental effects on the cerebral perfusion driven by presence or absence of regular physical activity as brain-vessel tortuosity decreases cerebral flow.

Given the short duration of the study phase (6 months) and the young age of our participants, we consider it rather unlikely that the decrease in perfusion observed in the CG is caused by a veritable age-related decline. A possible factor to be considered could be lipid metabolism. It is known that a sedentary life-style correlates positively with increased LDL–cholesterol levels (Crichton and Alkerwi, 2015). In turn, higher levels of cholesterol were shown to be associated with decreased regional perfusion in the superior temporal lobe (Claus et al., 1998). Unfortunately, we have no measures to verify these putative mechanisms and this will need to be assessed in future randomized exercise interventions.

Outside the temporal lobe, we observed that higher perfusion in the precentral gyrus correlated positively with individual VO_2max_. The results suggest vascular plasticity of the motor system in an anatomically restricted area that is precisely confined to the relevant leg representation of the primary motor cortex. The results are consistent with the concepts of promoting motor function through movement, as proposed by Perrey (2013). More recently, somatotopically restricted changes in the leg representation of the primary motor cortex after exercise have been described using quantitative longitudinal relaxation rate (R1) MRI, where R1 reflects cortical myelin density (Rowley et al., 2020). These data from healthy older adults (age range of 65–90 years) who exercised three times per week or maintained their usual physical routine over a 12-week period suggest plasticity at the microstructural level. Similar to our results, the effects correlated with a marker of improvement in submaximal aerobic performance (Rowley et al., 2020). Our results are also consistent with work in adult rats trained with the running wheel for 30 days, indicating capillary growth in motor areas of the cerebral cortex as an adaptation to sustained motor activity (Swain et al., 2003). In addition to capillary growth, the vasculature showed increased flow under conditions of activation (Swain et al., 2003). In endurance runners, greater gray matter volume and cortical surface area were reported in the left precentral gyrus compared to controls (Cao et al., 2021). Thus, bringing these non-human and human data together, they imply PE-induced locoregional plasticity in the primary motor cortex and suggest that perfusion is increased to meet increased energy demand as a long-term effect of PE.

Furthermore, bilateral occipital lobe perfusion correlated positively with VO_2max_. The occipital lobe was described in previous cross-sectional work comparing sedentary (10 men and 16 women, 54 ± 1 years) and endurance-trained (11 men and 21 women, 52 ± 1 years) middle-aged adults. Less carotid artery stiffness was associated with better neuropsychological outcomes, independent of age, sex, and education, and correlated with greater occipitoparietal blood flow (Tarumi et al., 2013).

Finally, we observed vascular plasticity in areas related to exercise-induced reward mechanisms. The association between VO_2max_ and ventral striatal perfusion in the IG is a novel finding, as this region has not typically been related to fitness-related outcomes. The ventral striatum, and especially dopaminergic signaling within the nucleus accumbens, has traditionally been associated with reward-related functioning, for example, motivational energization of behavior and reinforcement learning processes (Haber and Knutson, 2010; Salamone and Correa, 2012). In line with this reasoning, clinical studies observed that reductions in ventral striatal blood flow were correlated with apathy in psychiatric patients (Schneider et al., 2019). In this context, it is interesting that a recent observational study in *N* = 111 young adult women (Gorrell et al., 2022) found positive correlations between self-reported exercise engagement energization and brain responses during BOLD fMRI reward paradigms in the medial orbitofrontal cortex and, slightly weaker, in the ventral striatum. It is tempting to speculate whether regular engagement in vigorous physical exercise training that effectively increases VO_2max_ is also linked with upregulations in the motivational circuits of the brain, which would be consistent with findings from animal literature (Ruiz-Tejada et al., 2022), but needs more systematic research in humans. Consistent with our temporal and ventral striatal findings, Robinson et al. (2018) reported elevated resting brain glucose uptake in young and old healthy adults measured using ^18^F-fluorodeoxyglucose positron emission tomography after 12 weeks of high-intensity interval training (HIIT) training in parietal–temporal and caudate regions.

The present study convincingly shows that cortical perfusion increases can also be achieved by training interventions in young sedentary adults. We did not observe training-induced changes in hippocampal perfusion or associations between fitness and hippocampal CBF, as reported by previous studies in children (Chaddock-Heyman et al., 2016) and elderly populations (Burdette et al., 2010; Maass et al., 2015). This may have methodological reasons, but could also reflect developmental differences (Aghjayan et al., 2021), i.e., training effects on resting-state perfusion may be stronger during more dynamic phases of brain development and aging (or neurodegeneration). The available evidence from randomized controlled trials does suggest age-related differences regarding mechanisms and effects of exercise on brain health and cognition (reviewed in Stillman et al., 2020), although there are substantial gaps, especially in the research with young populations that preclude firm conclusions.

This study is not without limitations. We must acknowledge that the small sample size, particularly of the control cohort, may have limited the power of this study. Future studies should attempt to recruit larger samples of control subjects. Furthermore, the inactive control group, receiving the recommendation to maintain their usual (e.g., inactive) habits, might be a limitation of the study, as the missing attention, social contact, or light (physical) activities could have beneficial effects by themselves and may improve compliance. Moreover, we have to acknowledge that we did not quantify the entire daily physical activity in the CG. Rather, the activity tracker was used to rule out any deliberate physical activity violating the volunteer’s assignment to a passive control condition over a 6-month period. Ideally, future exercise studies should perform pCASL examinations at the same time of the day to rule out potential confounds due to diurnal variations of cerebral perfusion. For an in-depth review of additional external factors which may induce variation in cerebral blood flow measurements, empirical evidence for or against their significance and practical measures to minimize their influence, see Clement et al. (2018).

In summary, our randomized exercise intervention in sedentary young adults yielded region-specific increases in local perfusion that extend previous data from cross-sectional studies and provide a new database for younger cohorts. Interestingly, vascular plasticity in temporal, ventral striatal, and motor areas correlated with individual performance increases, suggesting a dependence on the exercise dose. These robust associations found with perfusion metrics are consistent with the literature showing positive effects of a physically active lifestyle on vascular health. The presented data support further efforts in initiating and maintaining physical exercise training in sedentary young individuals, as this might potentiate long-term beneficial health outcomes.

## Data availability statement

The original contributions presented in this study are included in the article/Supplementary material, further inquiries can be directed to the corresponding author.

## Ethics statement

The studies involving human participants were reviewed and approved by Ethikkommission an der Medizinischen Fakultät der Rheinischen Friedrich-Wilhelms-Universität Bonn. The patients/participants provided their written informed consent to participate in this study.

## Author contributions

NU and TSc performed statistical analysis, interpreted the data, and drafted and edited the manuscript. AM was involved in all aspects of project administration, investigation, and supervision, as well as reviewed and edited the manuscript. JC performed statistical analysis. LS and MD were involved in the conception and investigation of the project, reviewed, and edited the manuscript. JM was involved in the conception and investigation of the project, supervised the physical training and exercise tests, reviewed, and edited the manuscript. RS and TSt developed and supervised the in-house pCASL sequence, reviewed, and edited the manuscript. AR and UA contributed to provision of the infrastructure and critically revised the manuscript. HB was involved in the conception of the study and all aspects of the investigation, drafted, edited, and reviewed the manuscript. All authors contributed to the article and approved the submitted version.

## Footnotes

1
https://fsl.fmrib.ox.ac.uk/fsl/fslwiki/oxford_asl

2
https://www.nitrc.org/projects/mricrogl/

## Supplementary material

Supplementary Figure 1 Regression analyses between VO_2max_ and relative perfusion of **(A)** right superior temporal gyrus (rSTG), **(B)** right middle occipital region (rMOR), **(C)** right precentral gyrus (rPG), **(D)** right ventral striatum including the right lentiform nucleus (rVS), **(E)** rMOR, **(F)** left middle occipital region (lMOR), and **(G)** rVS. Part **(A–D)** show regressions pooled over all subjects (intervention group and control group) and all timepoints (T0, T2, T4, and T6), while **(E–G)** show regressions of all timepoints within the intervention group only.
