# Supplementary figures and images for: Regional cortical perfusion increases induced by a 6-month endurance training in young sedentary adults
Source: Front Aging Neurosci. 2022 Aug 9;14:951022. doi: 10.3389/fnagi.2022.951022 (PMC9407250; doi:10.3389/fnagi.2022.951022)

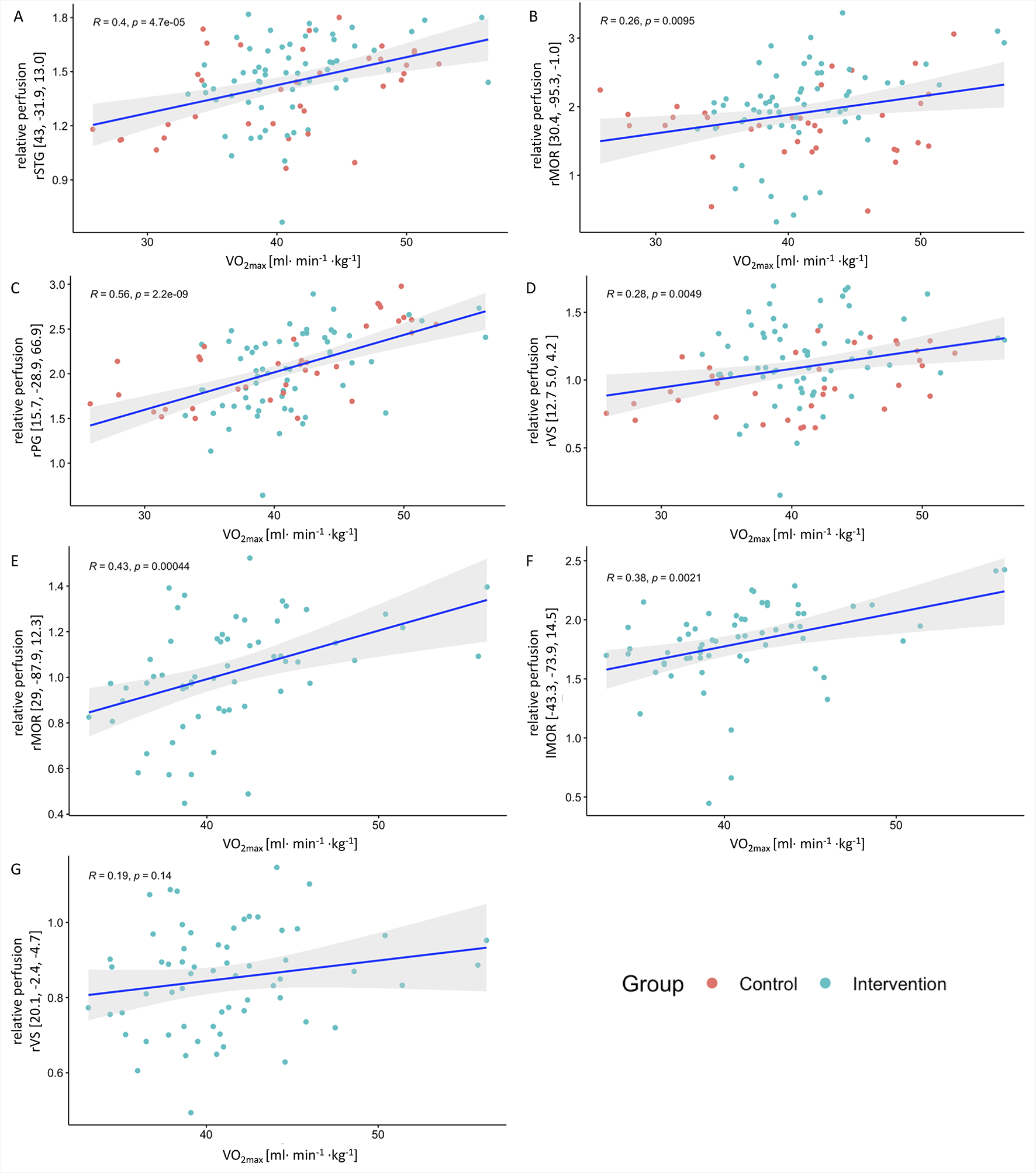

Supplement: Supplementary Figure 1 — Regression analyses between VO2max and relative perfusion of (A) right superior temporal gyrus (rSTG), (B) right middle occipital region (rMOR), (C) right precentral gyrus (rPG), (D) right ventral striatum including the right lentiform nucleus (rVS), (E) rMOR, (F) left middle occipital region (lMOR), and (G) rVS. Part (A–D) show regressions pooled over all subjects (intervention group and control group) and all timepoints (T0, T2, T4, and T6), while (E–G) show regressions of all timepoints within the intervention group only. [file Image_1.TIFF]
