# Supplementary material for: Regional cortical perfusion increases induced by a 6-month endurance training in young sedentary adults
Source: Front Aging Neurosci. 2022 Aug 9;14:951022. doi: 10.3389/fnagi.2022.951022 (PMC9407250; doi:10.3389/fnagi.2022.951022)
Supplement: Supplementary file 2 [file Table_1.DOCX]

Supplementary Table1: Global gray matter and white matter perfusion values in both control group (CG) and intervention group (IG) across all four time points (T0, T2, T4, T6).

| **ID** | **Group** | **GM_T0** | **WM_T0** | **GM_T2** | **WM_T2** | **GM_T4** | **WM_T4** | **GM_T6** | **WM_T6** |
| --- | --- | --- | --- | --- | --- | --- | --- | --- | --- |
| Subject01 | CG | 46.8 | 22.8 | 60.4 | 29.5 | 49.8 | 23.5 | 56.5 | 27.5 |
| Subject02 | CG | 62.9 | 28.7 | 64.5 | 29.3 | 59.3 | 27.1 | 60.0 | 26.6 |
| Subject03 | CG | 44.9 | 21.0 | 39.8 | 18.0 | 42.2 | 19.9 | 48.0 | 20.1 |
| Subject04 | CG | 54.7 | 25.3 | 44.4 | 21.4 | 47.2 | 22.2 | 40.3 | 19.4 |
| Subject05 | CG | 46.9 | 22.9 | 42.9 | 20.6 | 48.0 | 22.5 | 42.5 | 29.7 |
| Subject06 | CG | 43.7 | 21.7 | 48.2 | 21.8 | 47.1 | 20.2 | 45.6 | 20.4 |
| Subject07 | CG | 59.3 | 30.5 | 54.3 | 27.3 | 47.1 | 23.3 | 53.8 | 25.9 |
| Subject08 | CG | 46.8 | 22.9 | 57.1 | 26.7 | 61.2 | 27.9 | 61.6 | 28.7 |
| Subject09 | CG | 49.5 | 23.8 | 54.7 | 26.2 | 61.7 | 29.9 | 51.8 | 25.6 |
| Subject10 | CG | 39.7 | 19.6 | 31.0 | 16.3 | 42.7 | 20.4 | 37.3 | 16.9 |
| Subject11 | IG | 56.6 | 28.6 | 55.7 | 26.2 | 48.9 | 24.8 | 66.4 | 32.3 |
| Subject12 | IG | 63.7 | 29.0 | 53.8 | 27.6 | 45.4 | 22.1 | 50.3 | 24.4 |
| Subject13 | IG | 55.3 | 25.7 | 65.3 | 31.1 | 55.3 | 24.9 | 53.9 | 24.8 |
| Subject14 | IG | 58.6 | 28.9 | 57.7 | 28.8 | 56.6 | 26.0 | 53.8 | 25.4 |
| Subject15 | IG | 49.4 | 25.9 | 42.3 | 24.0 | 54.3 | 27.2 | 50.9 | 25.4 |
| Subject16 | IG | 69.9 | 34.4 | 73.7 | 34.7 | 68.8 | 32.3 | 66.4 | 30.8 |
| Subject17 | IG | 61.0 | 28.9 | 51.9 | 22.4 | 53.1 | 25.5 | 47.9 | 21.8 |
| Subject18 | IG | 56.9 | 28.1 | 47.1 | 25.8 | 49.0 | 24.2 | 55.9 | 26.4 |
| Subject19 | IG | 69.2 | 30.5 | 72.6 | 31.7 | 60.7 | 26.6 | 43.6 | 29.5 |
| Subject20 | IG | 52.2 | 26.3 | 51.2 | 25.7 | 73.2 | 36.3 | NA | NA |
| Subject21 | IG | 39.2 | 19.0 | 40.9 | 19.8 | 42.9 | 18.5 | 37.4 | 17.2 |
| Subject22 | IG | 57.5 | 28.3 | 22.7 | 20.6 | 56.7 | 28.8 | 53.7 | 25.3 |
| Subject23 | IG | 62.7 | 27.4 | 54.1 | 25.0 | 58.3 | 26.1 | 53.6 | 22.9 |
| Subject24 | IG | 44.8 | 22.9 | 54.9 | 28.1 | 66.4 | 33.5 | 56.4 | 30.3 |
| Subject25 | IG | 51.2 | 25.3 | 67.7 | 33.2 | 54.3 | 25.8 | NA | NA |
| Subject26 | IG | 48.5 | 24.5 | 45.2 | 24.6 | 45.7 | 23.0 | 43.6 | 21.9 |
| Subject27 | IG | 43.0 | 21.4 | 41.3 | 20.1 | 43.2 | 21.1 | 45.1 | 22.2 |
| Subject28 | IG | 38.9 | 20.2 | 50.2 | 23.8 | 37.3 | 16.9 | 37.3 | 15.9 |

GM=gray matter; WM=white matter; T0=baseline; T2= after two months; T4= after four months; T6=after six months.

|  | Intervention group (n=18)  mean ± SD | Control group (n=10)  mean ± SD | p-value |
| --- | --- | --- | --- |
| Global_GM_perfusion  T0 | 54.3 ± 9.0 | 49.5 ± 7.2 | 0.7051 |
| T2 | 53.3±12.4 | 49.7±10.3 | 0.963 |
| T4 | 53.9±9.2 | 50.6±7.4 | 0.934 |
| T6 | 51.7±8.7 | 49.7±8.3 | 0.999 |
